# Echinococcosis Is Associated with the Increased Prevalence of Intestinal *Blastocystis* Infection in Tibetans and Host Susceptibility to the *Blastocystis* in Mice

**DOI:** 10.3390/biology11050773

**Published:** 2022-05-18

**Authors:** Yang Zou, Yu-Gui Wang, Zhong-Li Liu, Ai-Jiang Guo, Xiao-Lu Li, Zhi-Qi Shi, Xing-Quan Zhu, Xiu-Min Han, Shuai Wang

**Affiliations:** 1 State Key Laboratory of Veterinary Etiological Biology, Lanzhou Veterinary Research Institute, Chinese Academy of Agricultural Sciences, Lanzhou 730046, China; zouyangdr@163.com (Y.Z.); wyg18894032073@outlook.com (Y.-G.W.); liuzl0219@163.com (Z.-L.L.); guoaijiang@caas.cn (A.-J.G.); lixiaolu07162022@163.com (X.-L.L.); shizhiqi903@163.com (Z.-Q.S.); 2 Laboratory of Parasitic Diseases, College of Veterinary Medicine, Shanxi Agricultural University, Taigu 030801, China; xingquanzhu1@hotmail.com; 3 Qinghai Clinical Research Institute of Hydatid Disease, Qinghai Provincial People’s Hospital, Xining 810007, China; 4 Jiangsu Co-Innovation Center for Prevention and Control of Important Animal Infectious Diseases and Zoonoses, Yangzhou 210009, China

**Keywords:** *Echinococcus multilocularis*, *Blastocystis*, prevalence, dual infection, host susceptibility

## Abstract

**Simple Summary:**

*Blastocystis* is a neglected enteric pathogen that is highly prevalent in humans and animals worldwide. Studies have reported that *Blastocystis* infection frequently coexists with other infectious pathogens in humans. However, dual infection by *Blastocystis* and *Echinococcus* *multilocularis*, which causes the severe parasitic disease echinococcosis has not been reported. In this study, the authors investigated the clinical prevalence, risk factors, and genotypes of *Blastocystis* in Tibetan patients with liver echinococcosis and Tibetan healthy controls from the Qinghai province in China, and also tested whether *E. multilocularis* infection increases host susceptibility to *Blastocystis* using a mouse model. The results found a significantly higher prevalence of genetically divergent *Blastocystis* in Tibetans with liver echinococcosis. *E. multilocularis* infection in Balb/c mice increased the host susceptibility to *Blastocystis* and aggravated intestinal pathology with higher disease severity and higher mortality. Taken together, these findings provide new insights into dual infections by *Blastocystis* and helminths in humans.

**Abstract:**

*Blastocystis* is a common human intestinal protozoan parasite. Little is known about its prevalence in echinococcosis. This study tested whether *Echinococcus multilocularis* infection would increase host susceptibility to *Blastocystis*. A total of 114 fecal samples (68 hydatid disease patients and 46 healthy people) were collected from Tibetans in the Qinghai province in China. The presence of *Blastocystis* was identified by sequencing of the small subunit (SSU) rRNA gene. Balb/c mice were co-infected with *Blastocystis* and *E. multilocularis* and tested for host susceptibility to *Blastocystis*. The overall *Blastocystis* prevalence was 12.3%; 16.2% in the patients and 4.4% in healthy people (*p* < 0.05). Sequence analysis identified three known *Blastocystis* genotypes, including ST1, ST2, and ST3, and one unknown genotype. Experimental dual infection significantly reduced mouse survival rate (20%), induced more severe signs, and increased intestinal damages with a higher intestinal colonization level of *Blastocystis*. The mouse model showed that *E. multilocularis* infection increases host susceptibility to *Blastocystis*. Our study shows a significantly higher prevalence of *Blastocystis* in patients with liver echinococcosis and reveals that non-intestinal *E. multilocularis* infection increases host susceptibility to the *Blastocystis*. Our results highlight that *E. multilocularis* infection is associated with *Blastocystis*. These findings remind us that more attention should be paid to the gut health of the patients with a helminth infection during clinical patient care.

## 1. Introduction

*Blastocystis* is one of the most common intestinal organisms found in humans worldwide. This parasite is genetically diverse with at least 22 identified valid genotypes, 10 of which (ST1-ST9 and ST12) have been reported in humans and animals [1,2,3]. Hosts are usually infected with *Blastocystis* via the fecal–oral route through the ingestion of contaminated water or food [4,5]. Today, *Blastocystis* is a common part of the healthy gut microbiota [6,7]. However, intestinal symptoms may emerge in presence of *Blastocystis,* and thus it may be considered pathogenic when other agents are eliminated [8]. Infection with *Blastocystis* has been reported to coincide with other parasitic diseases, and to be more common in patients with immune deficiency or chronic immunosuppression than in healthy people [9,10]. Chronic helminth infections typically induce suppression of host immunity [11]. Thus, helminth diseases can potentially increase the risk of *Blastocystis* infection in the gut. However, we still know little about dual infection prevalence and causal mechanisms with *Blastocystis* and helminths. Moreover, whether a chronic helminth infection can exacerbate *Blastocystis* infection pathogenesis remains unknown.

Among the most severe of the zoonotic helminth diseases, hydatid disease (echinococcosis), which includes alveolar echinococcosis (AE) and cystic echinococcosis (CE), represents a substantial disease burden; globally, AE and CE are the second and third most important food-borne parasitic diseases, respectively [12]. The worldwide prevalence of hydatid disease is estimated at 1 million with an annual incidence of 200,000 [13]. Each year, hydatid disease is estimated to claim 19,300 lives and result in around 871,000 disability-adjusted life years (DALYs) globally (WHO FERG, 2015). The Chinese Qinghai province (hereon Qinghai) belongs to the Qinghai–Tibet Plateau region located in western China, which is the main epidemic region for human echinococcosis around the world.

The main aims of our study were to investigate the prevalence of *Blastocystis* in patients with hydatid diseases from the Qinghai Tibetans population and explore whether helminth infection increases host susceptibility to *Blastocystis* infection. This work will help provide scientific support to develop better preventive and control strategies in dual infections between *E. multilocularis* and *Blastocystis*.

## 2. Materials and Methods

### 2.1. Sample Collection

Fresh fecal samples were collected from 114 people recruited from the Qinghai People’s Hospital. These included 68 patients with liver echinococcosis and 46 healthy individuals. A diagnosis of liver echinococcosis was based on HD-specific IgG ELISA, ultrasonographic features, computed tomography, and surgical findings. Healthy controls did not have liver echinococcosis as determined by negative tests for hydatid antibodies. Individuals with chronic diseases (such as cancers and diabetes), chronic viral diseases, and those who had used antiparasitic medications within the last 3 months were excluded. The fresh fecal samples were stored at −80 °C immediately after collection and kept frozen until DNA extraction to test whether echinococcosis would increase host susceptibility to *Blastocystis*. The fecal DNA was extracted from inpatients with liver echinococcosis and 46 healthy individuals were examined for the positivity of *Blastocystis*. In addition, venous blood was collected from 68 patients with liver echinococcosis and routine clinical tests were performed to test for an association with *Blastocystis* infection. The blood specimens were collected from 68 patients with liver echinococcosis from January 2021 to December 2021. The blood routines were retrieved from the diagnosis step at admission. Stool samples for each participant were also collected at the same time.

### 2.2. Mouse Model

We used 7–9-week-old female-specific pathogen-free (SPF) Balb/c mice (females) purchased from the Laboratory Animals Center of Lanzhou Veterinary Research Institute, Chinese Academy of Agricultural Sciences. A total of 14 Balb/c mice were divided into four groups, including (1) a dual infection group (n = 5) that was infected with *E. multilocularis* (2000 protoscolex per mouse) and *Blastocystis* ST1 strain (1 × 10^5^ cells per mouse), (2) a *Blastocystis* infection group (n = 3) that was infected with the ST1 strain (1 × 10^5^ cells per mouse), (3) an *E. multilocularis* infection group (n = 3) that was infected with *E. multilocularis* protoscolex (n = 2000), and (4) a control group (n = 3) that was treated with phosphate-buffered saline (PBS). Mice were inoculated with *E. multilocularis* using the *E. multilocularis* protoscolex (n = 2000) from hydatid cysts collected from a Mongolian gerbil (*Meriones unguiculatus*) that had been infected with *E. multilocularis* for five months. The protoscolex was collected and filtered through an 80-mesh copper mesh, followed by two washes with PBS supplemented with 1% penicillin/streptomycin. We used the ST1 *Blastocystis* strain to infect our mice as it has been demonstrated to cause an asymptomatic phenotype in rodents [14]. The *Blastocystis* strain (ST1) was a gift from Dr. Lei Ma at Hebei Normal University. Mice were infected with either the *E. multilocularis* protoscolex or treated with PBS depending on group assignment. The dual infection and *Blastocystis* infection groups were gavaged with *Blastocystis* (1 × 10^5^ cells per mouse). The *E. multilocularis* infection and negative control mice were euthanized after three months, and their intestinal tissues and lamina propria cells were collected for histopathological staining and T lymphocyte isolation, respectively. After infection with *Blastocystis*, feces from the dual infection and *Blastocystis* infection mice were collected every two days and used to detect *Blastocystis* colonization. The dual infection and *Blastocystis* infection mice were euthanized on day 14 post infection. The feces and intestinal tissues of the mice were collected and used for DNA extraction and histopathological staining, respectively. The tissue was fixed in 4% paraformaldehyde for 48 h, dehydrated, and then embedded in paraffin. Slices were dewaxed, rehydrated, and stained with hematoxylin-eosin. The symptoms of piloerection and torpidity were estimated by observation of “absence” and “presence”.

### 2.3. T Lymphocyte Isolation

Flow cytometry analyses of T lymphocytes in the lamina propria of the small intestine were performed in samples taken from mice infected with either 2000 *E. multilocularis* protoscolex or PBS on day 90 post infection, according to the protocol described in a previous study [15]. Briefly, intestines were harvested from mice, cut open longitudinally, and washed in PBS. The fat tissues and Peyer’s Patches (PPs) were removed. Intestines were then cut into 2 cm pieces and washed on a shaker in PBS containing 1 mM DTT for 10 min at 37 °C. After that, the intestines were incubated twice with shaking in PBS containing 30 mM EDTA at 37 °C for 10 min; the fluid was replaced between cycles. Then, the intestines were further cut into 0.5 cm pieces. The tissues were then digested with shaking in RPMI1640 medium (Gibco, Waltham, MA, USA) containing DNase I (Solarbio, Beijing, China) (150 μg/mL) and collagenase VIII (Gibco, Waltham, MA, USA) (200 U/mL) at 37 °C for 70 min. The digested tissues were homogenized by vigorous shaking and then passed through a 70 μm cell strainer to remove large debris. The flow-through was centrifuged in a Percoll gradient at 800× *g* for 20 min at room temperature and the mononuclear T lymphocytes were harvested from the 40%/80% interphase. Flow cytometry was performed to analyze regulatory T cells (Treg) according to a previous study [16].

### 2.4. Genomic DNA Extraction and PCR Amplification

Approximately 200 mg of fecal sample was extracted for genomic DNA using the E.Z.N.A. Stool DNA kit (OMEGA, Norcross, Georgia) and eluted into a final volume of 100 μL according to the manufacturer’s protocol. Genomic DNA from each sample was stored at −20 °C for further PCR amplification. *Blastocystis* positivity was screened by PCR targeting a fragment of the SSU rRNA with primers (RD5: 5′-ATCTGGTTGATCCTGCCAGT-3′ and BhRDr: 5′-GAGCTTTTTAACTGCAACAACG-3′) [17]. The 25 μL reaction system consisted of 2 μL genomic DNA, 0.2 mM dNTP mixture, 1.5 mM MgCl_2_, 2.5 μL of 10 × buffer, 1.25 U of TaKaRa Ex Taq^®^ (Takara Dalian, China), and 0.25 μL of primers (10 mol/μL). The PCR reaction conditions were set as follows: initial denaturation at 94 °C for 5 min, 35 cycles including 94 °C for 45 s, 59 °C for 45 s, and 72 °C for 1 min; finally, an additional 72 °C extension for 3 min. Each PCR reaction included negative and positive controls.

### 2.5. DNA Samples for Quantitative Real-Time PCR Assay

For Quantitative Real-time PCR (qPCR) analysis, all the fecal DNA in the mice was standardized to 100 ng/μL. The qPCR amplifications were performed using an Applied Biosystems (ABI) 7500 Real-Time PCR system (Thermo Fisher Scientific, Waltham, MA, USA) in a 20 μL reaction volume containing 10 μL of 2x GoTaq@ qPCR Master Mix (Promega Corporation, San Luis Obispo, CA, USA), 3.65 mM MgCl_2_, 0.2 μM of each primer (BL18SPPF1: 5′-AGTAGTCATACGCTCGTCTCAAA-3′ and BL18SR2PP: 5′-TCTTCGTTACCCGTTACTGC-3′) [18], and 3 μL of DNA template. The qPCR reaction consisted of pre-denaturation at 95 °C for 3 min and 35 cycles for 45 s of denaturation at 95 °C, 45 s of annealing at 65 °C, and 1 min of extension at 72 °C. For normalization, genomic DNA (Blastocystis-positive DNA) from an in vitro culture of *Blastocystis* strain (ST1) was used to establish a standard curve line for DNA concentration of *Blastocystis* according to the method in the literature [18]. A Ct value < 35 was considered positive [19]

### 2.6. Phylogenetic Groups of Blastocystis

The PCR-positive products were subject to DNA sequencing in the TSINGKE Bio-logical Technology Company (Xian, China). Obtained sequences were checked with their DNA peak form graph by Chromas v.2.6, and the genotypes of Blastocystis were identified by aligning the sequences with the corresponding genotype sequences retrieved from the GenBank database (http://www.ncbi.lm.nih.gov/GenBank/ accessed on 26 July 2021). The sequences of the Blastocystis were aligned with the Clustal W algorithm using MEGA 7 (http://www.megasoftware.net/ accessed on 26 July 2021). Because the nucleotide sequences of Blastocystis used in this study differ in length, ends of sequences were trimmed by Clustal X v.2.0. The phylo-genetic analyses were performed using maximum likelihood (ML) methods (Ki-mura-2-parameter model with 1000 bootstrap replicates) implemented in MEGA7 to infer the genetic relationships.

### 2.7. Statistical Analysis

Statistical analysis for the prevalence of *Blastocystis* was performed using chi-squared tests in SPSS 24.0 (SPSS Inc., Chicago, IL, USA). The 95% confidence intervals (CIs) were calculated. Odds ratios (OR) with 95% confidence intervals (CI) were used to identify risk factors of *Blastocystis* infection. The difference was considered significant when *p* < 0.05.

## 3. Results

### 3.1. Increased Prevalence of Blastocystis Infection in Patients with Hydatid Diseases

A total of 13 (11.4%, 95% CI: 5.57–17.24) of 114 samples were positive for *Blastocystis* infection. Tibetan females (18.0%) were marginally more susceptible to infection with *Blastocystis* than males (6.3%, *p* = 0.05). Additionally, the highest prevalence of *Blastocystis* was observed in older Tibetans aged over 65 years (33.3%), followed by 10.5% in Tibetans aged 18–65 years, and 7.7% in those aged less than 18 years (Table 1). Notably, the prevalence of *Blastocystis* in hydatidosis patients (16.2%) was significantly higher than that in healthy individuals (4.4%) (*p* < 0.05) (Table 2). Routine blood tests and biochemical indices were used to test for associations with *Blastocystis* in hydatidosis patients. We found that lipase (LPS) and total bilirubin (TBIL) were associated with the prevalence of *Blastocystis* in these patients, and the odds ratios were 3.9 and 6.1, respectively (Table 3). These results indicate that liver hydatid disease is associated with a higher rate of intestinal *Blastocystis* infection in Tibetans.

### 3.2. Genotyping and Phylogenetic Analysis

**Figure 1:**
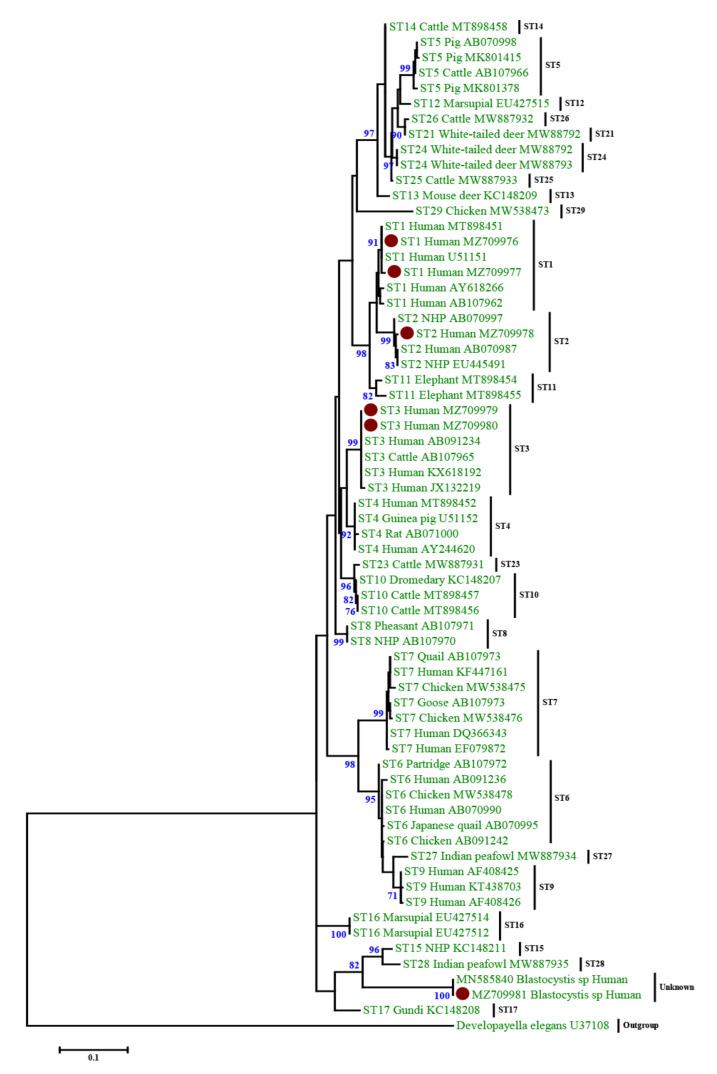
Phylogenetic analysis of Blastocystis using maximum likelihood method based on the SSU rRNA gene sequences. Developayella elegans was used as outgroup taxon to root the tree. Analysis was conducted by a maximum likelihood method. Genetic distances were calculated using the Kimura two-parameter model. This analysis involved 66 nucleotide sequences. Bootstrap values lower than 60% are not displayed. The Blastocystis sequences determined in this study are indicated with a red circle.

Using SSU rRNA sequence analysis, we identified four genotypes of *Blastocystis* in the 13 *Blastocystis*-positive samples, including three known genotypes, ST1 (n = 6, 46.15%), ST2 (n = 1, 7.69%), ST3 (n = 5, 38.46%), and one unknown genotype (n = 1, 7.69%). Genotype sequences were used to infer the phylogenetic relationships (Figure 1). The phylogenetic tree revealed that the sequences of *Blastocystis* from this study were highly similar to those of other *Blastocystis* isolates previously deposited in GenBank. Moreover, no mixed infections of *Blastocystis* were detected in any of the examined samples.

### 3.3. E. multilocularis Infection Increases Host Susceptibility to Blastocystis in a Balb/c Mouse Model

**Figure 2:**
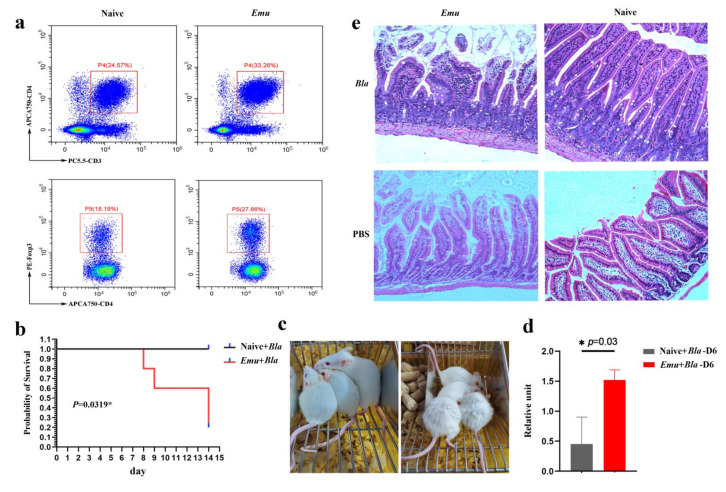
*E. multilocularis* (*Emu*) infection increased the susceptibility to *Blastocystis* (*Bla*) in a mouse model. (**a**) Flow cytometry analysis revealed that regulatory T cells (Treg) were expanded in the Balb/c mice that were pre-infected with *E. multilocularis* 3 months ago. (**b**) The survival rate of mice with dual infection (*E. multilocularis* and *Blastocystis*) and single infection (*Blastocystis*). (**c**) Symptoms of mice with a single infection (left) and with dual infection (right). The piloerection and torpidity were observed in mice with dual infection (right) but not in the mice with a single infection (left). (**d**) qPCR detection of *Blastocystis* in feces of the mice at day 6 post-infection. (**e**) H&E staining of jejunum in for the naive mice, and the mice with single (*E. multilocularis* or *Blastocystis*) or dual infection (*E. multilocularis* and *Blastocystis*). The * represents the difference was statistically significant.

To confirm whether liver hydatid disease increases host susceptibility to *Blastocystis* infection, we used a mouse model that can be colonized by a human ST1 isolate (Figure S1). Balb/c mice were pre-infected with *E. multilocularis*, which is a causative agent for liver hydatid disease. Consistent with the findings in the previous studies that chronic *E. multilocularis* infection typically suppresses host immunity [20], we also observed an expansion of regulatory T cells (Treg) (Figure 2a), suggestive of an immune state of suppression. After inoculation with *Blastocystis* (ST1 isolate), 80% of the mice in the dual infection group died within 14 days post infection. Conversely, all control mice survived following *Blastocystis* infection (Figure 2b). Moreover, Balb/c mice that had been pre-infected with *E. multilocularis* developed a more severe illness, with typical symptoms of piloerection and torpidity, than the mice that were only infected with *Blastocystis* (Figure 2c). The qPCR quantification of fecal *Blastocystis* indicated that the colonization was significantly higher in the dual infection group than in control mice (Table S1). This suggests that *Blastocystis* colonized and proliferated more easily in the gut of the dual-infected mice (Figure 2d). Moreover, the mice singly infected with *E. multilocularis* and negative control mice did not show any detected level of *Blastocystis* in their feces (data not shown). H&E staining also showed more extensive pathology in the jejunum tissue in the dual infection mice (Figure 2e). Collectively, these results suggest that chronic *E. multilocularis* infection increases host susceptibility to gut pathogen colonization and aggravates the pathogenesis caused by *Blastocystis*.

## 4. Discussion

Previous studies have shown that *Blastocystis* can co-occur with protozoa [21,22], HIV [23], malignant tumor [24], tuberculosis [25], and urticaria [26] in humans. However, no cases of combined *Blastocystis* and hydatidosis have been reported. The prevalence of hydatid disease was 4.5%, 4.7%, and 1.2% in the Qinghai province in 2012, 2014, and 2018, respectively, which represents one of the most prevalent regions around the world [27,28,29]. In this study, we found a *Blastocystis* prevalence in Tibetans of 12.3% (95% CI: 5.57–17.24). The prevalence was significantly higher in patients with hydatidosis (16.2%) than in healthy individuals (4.4%), suggesting that the former have an increased susceptibility to *Blastocystis* infection. Our prevalence data for the healthy population agree with a comprehensive review by Zhang et al. in which the average infection rate of *Blastocystis* worldwide was 4.4% (20,236/457,501) [30]. The prevalence of *Blastocystis* is influenced by multiple epidemiological factors. Jiménez et al. have proposed that *Blastocystis* in humans is associated with poor conditions and poor access to clean drinking water [31]. Additional factors such as lifestyle and dietary habits, poultry or livestock farming, poor immune function, poor nutritional status, female sex, low body mass index < 19, anemia, and barefoot farm work are risks associated with *Blastocystis* infection in humans [32,33,34,35]. In this study, we found that almost all these factors were present in our participants who originate from the same region and share similar lifestyles and animal-based diets. We found that the prevalence of *Blastocystis* in females was marginally significantly higher than that in males (*p* = 0.05); this concurs with previously published data. Furthermore, there was no statistical difference in prevalence among age groups in the present study (*p* = 0.17).

By excluding these sex factors, the higher prevalence of *Blastocystis* found in hydatidosis patients is likely to be related to immunocompetence. Previous studies have reported a higher *Blastocystis* infection rate in patients with malignant tumors due to a weakened immune system [36,37]. Chronic *E. multilocularis* infection is marked by significant suppression of host immunity [22,38,39], which may provide a permissive environment for invasion by other pathogens. Indeed, *Blastocystis* infection is frequently observed in immunocompromised individuals, with a high reported prevalence of 15.0–72.4% [10]. Here, we speculate that hydatid disease increased the susceptibility to *Blastocystis* in our cohort. Interestingly, routine blood tests revealed marginal associations with *Blastocystis* infection; patients with an abnormal lipase index (OR = 3.90, 95% CI: 0.78–19.58) were more likely to infect with *Blastocystis* compared to those with a normal index. Such elevation of lipase is regularly observed during immune checkpoint inhibition (ICI) [40]. We speculate that the elevation seen in our cohort might reflect such immune suppression. Furthermore, immunocompromised individuals had a higher prevalence of *Blastocystis* [10], so the abnormal lipase index could also have been related to *Blastocystis* infection. In contrast, total bilirubin levels in hydatidosis patients were marginally associated with *Blastocystis* infection (OR = 6.11, 95% CI: 0.76–49.05). It has been demonstrated that serum bilirubin has substantial anti-inflammatory and anti-oxidative properties [41,42]. Blood levels of total bilirubin are also normally present in the gut and can cross the gut cell membranes [43]. Thus, bilirubin might protect the gut, resulting in a lower prevalence of *Blastocystis* in hydatidosis. The observed marginal association between blood routines and *Blastocystis* infection might reflect some cause or consequences, but no previous studies have reported this relationship. Thus, further research is needed to profoundly investigate it.

At least 22 valid genotypes (STs) of *Blastocystis* have been described among mammalian and avian isolates [1,5,30], of which nine genotypes (ST1-ST9 and ST12) are reported in the human population [4]. In Asia, the most prevalent genotypes include ST1, ST2, and ST3. In China, ST1-ST7, ST12, and unknown genotypes have also been identified in humans [30]. Consistent with these prior studies, we identified three genotypes, ST1 (n = 6), ST2 (n = 1), and ST3 (n = 5), and one unknown genotype (n = 1) in our cohort. The unknown genotype was clustered into a separate branch that is relatively far from the ST1, ST2, and ST3 and is more closely related to ST15 and ST28 (Figure 1).

To test whether *E. multilocularis* infection is able to increase host susceptibility to *Blastocystis*, we constructed an *E. multilocularis* and *Blastocystis* dual infection Balb/c mouse model. Consistent with previous findings that chronic Echinococcus infection modulates host immunity by inducing the proliferation of Treg cell proliferation [44,45], we found a significant expansion of CD4^+^ FoxP3^+^ Treg cells in the *E. multilocularis* infection model (Figure 2a); this indicates that immune suppression was induced by the helminth infection. This may explain why susceptibility to *Blastocystis* is increased in this model. In addition, we found that *Blastocystis* induced higher mortality with increased gut inflammation and more severe pathogenic damage in the intestinal tissue in mice that were pre-infected with *E. multilocularis* (Figure 2c,e), further suggesting that the host immunity was suppressed after *E. multilocularis*. Similarly, the level of *Blastocystis* infection in mouse feces was higher in the dual infection group (Figure 2d). A high level of *Blastocystis* infection has also been shown in HIV/AIDS patients in the Anhui province of China [46]. Together, our data suggest that the *E. multilocularis* infection could increase host susceptibility to *Blastocystis* and aggravate the resultant pathological damage in the gut.

## 5. Conclusions

In conclusion, to the best of our knowledge, this is the first study to report a higher co-occurrence of *E. multilocularis* and *Blastocystis* in the Tibetan population. Findings from our dual infection mouse model provide direct evidence that links non-intestinally parasitic helminth *E. multilocularis* infection and increased host susceptibility to the intestinal protozoan *Blastocystis*. Further work is needed to more clearly elucidate the mechanisms underlying this dual infection.

## Figures and Tables

**Table 1:** Prevalence and factors associated with *Blastocystis* infection in Tibetan people in Qinghai, China.

Factors	Category	Sample	No. Positive	% (95% CI)	OR (95% CI)	*p*-Value ^a^
Age	<18 yr	13	1	7.7 (0–22.18)	1	0.17
	18–65 yr	95	10	10.5 (4.36–16.70)	1.41 (0.16–12.03)	
	>65 yr	6	2	33.3 (0–71.05)	6.0 (0.42–85.25)	
Gender	Male	64	4	6.3 (0.32–12.18)	1	0.05
	Female	50	9	18.0 (7.35–28.65)	3.29 (0.95–11.41)	
Total		114	13	11.4 (5.57–17.24)		

^a^ chi-square test.

**Table 2:** Prevalence and factors associated with *Blastocystis* infection in hydatidosis patients and healthy people in Qinghai, China.

Factor	No. Tested	No. Positive	Prevalence (%) (95% CI)	OR (95% CI)	*x^2^ p*-Value ^lr^	*x^2^ p*-Value ^a^
Health	46	2	4.4 (0–10.24)	1	*p* = 0.039 ^lr^	*p* = 0.051
Hydatidosis patients	68	11	16.2 (7.42–24.93)	4.25 (0.90–20.15)		
Total	114	13	12.3 (6.26–18.31)			

OR: odds ratio; CI: confidence interval; ^lr^ likelihood ratio test; ^a^ chi-square test.

**Table 3:** Prevalence and factors associated with *Blastocystis* infection in blood routine and blood biochemical indexes of hydatidosis patients in Qinghai, China.

Variable	Category	No. Tested	No. Positive	Prevalence (%) (95% CI)	OR (95% CI)	*p*-Value ^a^
EO_Num	<0.02 or >0.52 ^ab^	62	10	16.13 (6.97–25.28)	1	0.97
	0.02–0.52 ^n^	6	1	16.67 (0–46.49)	1.04 (0.11–9.88)	
BASO_Num	0–0.06 ^n^	52	8	15.38 (5.58–25.19)	1	0.75
	>0.06 ^ab^	16	3	18.75 (0–37.87)	1.269 (0.29–5.49)	
LYMPH_Percent	<20 or >50 ^ab^	14	2	14.29 (0–32.62)	1	0.83
	20–50 ^n^	54	9	16.67 (6.73–26.61)	1.20 (0.23–6.31)	
LPS	13–60 ^n^	60	8	13.33 (4.73–21.93)	1	0.08
	>60 ^ab^	8	3	37.50 (3.95–71.05)	3.90 (0.78–19.58)	
TBIL	<5 or >21 ^ab^	64	9	14.06 (5.55–22.58)	1	0.06
	5–21 ^n^	4	2	50.00 (1.0–99.00)	6.11 (0.76–49.05)	

^a^ chi-square test; ^n^ normal; ^ab^ abnormal; EO_Num: number of eosinophils; BASO_Num: number of basophils; LYMPH_Percent: Percentage of lymphocytes; LPS: Lipase; TBIL: Total bilirubin. Only variables with abnormal values that account for more than 10% of the samples are shown.

## Data Availability

The datasets supporting the findings of this article are included within the article. The sequences obtained in the study are deposited in GenBank under the accession numbers: MZ709976-MZ709981 (https://submit.ncbi.nlm.nih.gov/subs/genbank/SUB10154998/overview/, accessed on 5 August 2021).

## Supplementary Materials

The following supporting information can be downloaded at: https://www.mdpi.com/article/10.3390/biology11050773/s1, Figure S1: PCR amplification of *Blastocystis* in mice feces; Table S1: The results of qPCR detection of *Blastocystis* in mice feces. COIN represents the dual infection group (*E. multilocularis* and *Blastocystis*), SIN represents a single infection group (*Blastocystis*). The Ct value < 35 was considered positive. Lane M represents DL2000-bp DNA marker, Lane A represents the PCR amplification product of *Blastocystis* in dual infection mice feces, Lane B/C/D represents the PCR amplification product of *Blastocystis* in the fecal samples of the mice with the single infection of *Blastocystis*, Lane E represents positive control of *Blastocystis* DNA, and Lane F represents native control for *Blastocystis* DNA.
