# Supplementary figures and images for: Echinococcosis Is Associated with the Increased Prevalence of Intestinal Blastocystis Infection in Tibetans and Host Susceptibility to the Blastocystis in Mice
Source: Biology (Basel). 2022 May 18;11(5):773. doi: 10.3390/biology11050773 (PMC9138466; doi:10.3390/biology11050773)

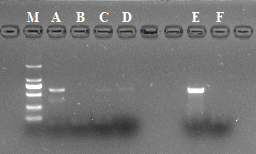

Supplement: Supplementary file 1 [file biology-11-00773-s001.zip › Figure. S1.tif]
